# Prospective randomized controlled trial of omental roll‐up technique on pancreatojejunostomy anastomosis for reducing perioperative complication in patients undergoing pancreatoduodenectomy

**DOI:** 10.1002/jhbp.948

**Published:** 2021-05-04

**Authors:** Pongsatorn Tangtawee, Somkit Mingphruedhi, Narongsak Rungsakulkij, Wikran Suragul, Watoo Vassanasiri, Paramin Muangkaew

**Affiliations:** ^1^ Hepato‐Pancreato‐Biliary Division Department of Surgery Faculty of Medicine Ramathibodi Hospital Mahidol University Bangkok Thailand

**Keywords:** omental roll‐up, omentum, pancreatic fistula, pancreatoduodenectomy, whipple operation

## Abstract

**Background:**

Wrapping pancreatojejunal anastomosis with omentum to prevent postoperative pancreatic fistula (POPF) has only been reported in non‐randomized, controlled trials. Therefore, this study aimed to conduct a randomized, controlled trial to compare outcomes between omental roll‐up and non‐omental roll‐up in pancreatojejunal anastomosis.

**Methods:**

This single‐center, randomized, two‐arm trail (Clinical Trials Register: NCT03083938) was conducted between February 2017 and February 2019. We studied 34 patients in the omental roll‐up group and 34 patients in the non‐omental roll‐up group. The primary endpoint was the incidence of clinically relevant POPF. Thirty‐day mortality and morbidity were recorded.

**Results:**

Patients’ demographic data were not significantly different between the two groups, except for histological diagnosis, with a significantly higher incidence of pancreatic cancer in the omental roll‐up group (n = 15, 44.1%) than in the non‐omental roll‐up group (n = 9, 26.4%) (*P* = 0.042). There was one death in the non‐omental roll‐up group due to myocardial infarction. The incidence of POPF was not different between the omental roll‐up group (n = 5, 14.7%) and non‐omental roll‐up group (n = 7, 20.6%) (*P* = 0.525). No differences were found in postoperative hemorrhage after pancreatectomy, delayed gastric emptying, and chyle leakage between the groups.

**Conclusion:**

This study shows that omental roll‐up does not decrease the incidence of POPF after pancreatoduodenectomy.

## Graphical abstract

**Highlight**

Tangtawee and colleagues conducted the first single‐center prospective randomized controlled trial of the omental roll‐up technique for pancreatojejunostomy to elucidate its potential benefit in reducing the incidence of postoperative pancreatic fistula in patients undergoing pancreatoduodenectomy. Omental roll‐up did not significantly reduce the incidence of clinically relevant pancreatic fistula after pancreatoduodenectomy.

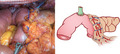

## 1 INTRODUCTION

Pancreatoduodenectomy (PD) is the standard treatment of periampullary tumors. However, the overall morbidity rate, including intra‐abdominal collection, hemorrhage post‐pancreatectomy, and delayed gastric emptying, are still high, with rates of approximately 65.9%‐77.5%.^1^, ^2^ The causes of these complications are usually from postoperative pancreatic fistula. Several methods have been attempted to reduce the incidence of pancreatic fistula after undergoing pancreatoduodenectomy, such as pancreatic stenting, use of intravenous somatostatin, use of sealant material, and wrapping anastomosis with soft tissue.^3^

Wrapping pancreatojejunal anastomosis with omentum is not a complicated procedure and requires no extra medical costs for the patient.^4^ This technique has been applied in non‐randomized, controlled trials (non‐RCTs), but their data did not show that this technique could significantly reduce the pancreatic fistula rate.^5^ Therefore, this study aimed to conduct an RCT to compare outcomes between omental roll‐up at pancreatojejunal anastomosis and non‐omental roll‐up at pancreatojejunal anastomosis.

## 2 METHODS

### 2.1 Data collection

**FIGURE 1:**
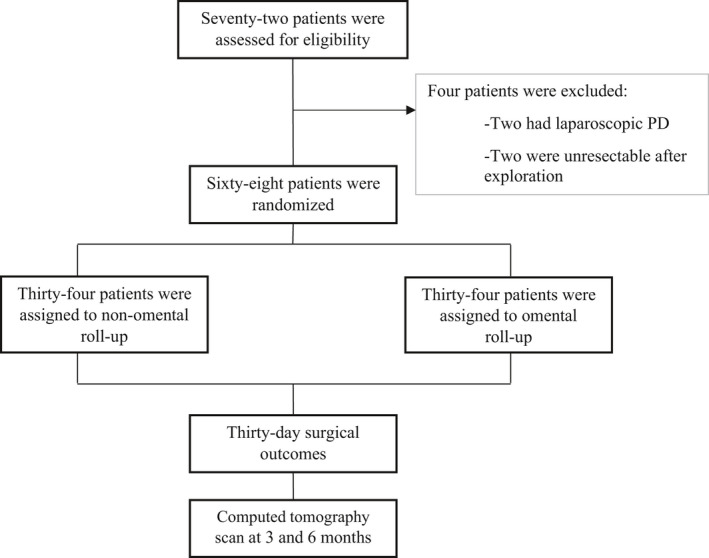
Diagram of the patients’ randomization and follow‐up

This single‐center, randomized, two‐arm trial was conducted between February 2017 and February 2019, and was approved by the ethics committee and registered (Clinical Trials Register: NCT03083938). Patients who were eligible for this study were scheduled for pancreatoduodenectomy. The inclusion criteria for the study were patients aged >18 years and patients who were scheduled for resectable PD. The exclusion criterion was patients who previously underwent omental resection (Figure 1). Randomization was generated by a computer program and sealed envelopes labels were opened in the operating room after pancreaticojejunal anastomosis was performed. The 30‐day mortality and morbidity rates were recorded. A follow‐up computed tomography (CT) scan was performed at 3 and 6 months for visualization of portal vein compression after omental roll‐up.

### 2.2 Surgical technique

The operations were performed by five surgeons. The cefoxitin was used as a preoperative prophylactic antibiotic and was continued for 24 hours postoperatively. After intra‐abdominal staging was performed, the classical Whipple's procedure or pylorus‐preserving pancreaticoduodenectomy (PPPD) was performed on the basis of extension of the tumor. Lymph nodes of the hepatoduodenal ligament, the common hepatic artery, and the right side of the superior mesenteric artery were dissected. Duct to mucosa was the first preferred choice. However, pancreatoenteric anastomosis was performed by the invagination technique if the main pancreatic duct could not be identified. A pancreatic duct stent was placed on the basis of the surgeon's preference and postoperative somatostatin was provided on the basis of the surgeon's preference after POPF occurred. Two surgical drains were placed at the peripancreatojejunal anastomosis. The drain was removed if amylase‐rich fluid was less than 5 mL/day. Amylase, bilirubin, and triglyceride concentrations of the drainage fluid were measured at postoperative days 1, 3, and 5.

### 2.3 Intervention

**FIGURE 2:**
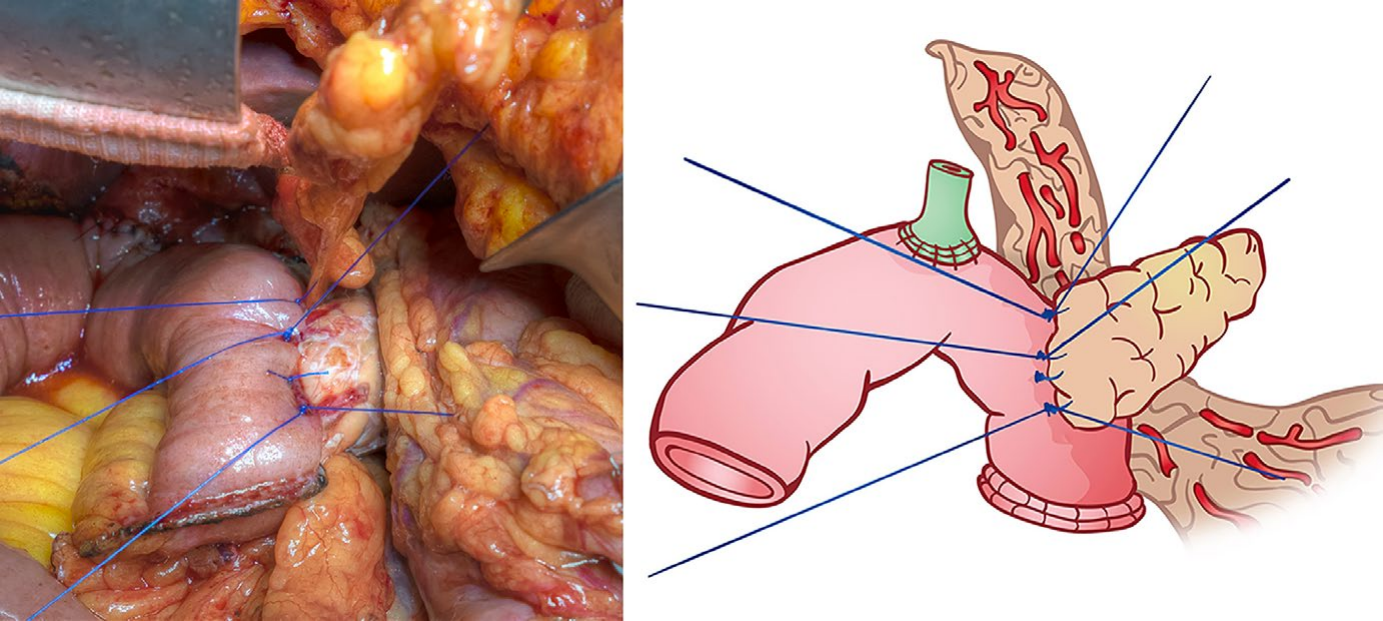
The omentum is brought to the posterior side of pancreatojejunal anastomosis

**FIGURE 3:**
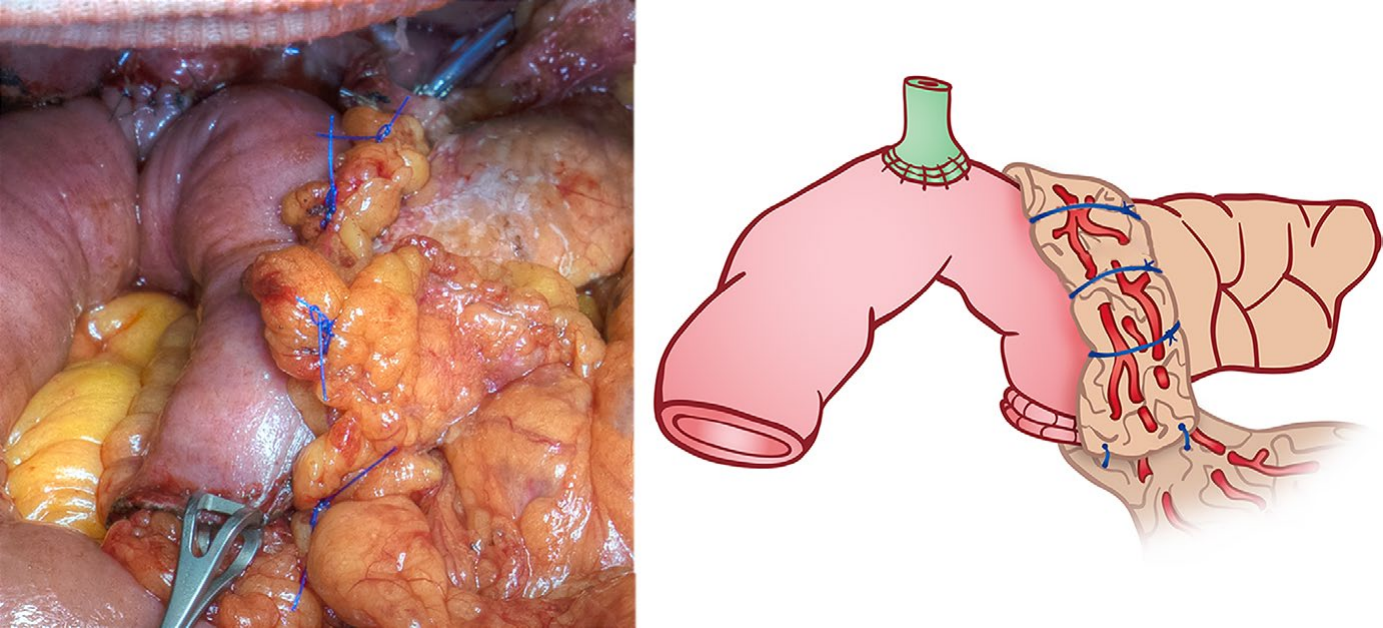
Omental wrapping and fixing at pancreatojejunal anastomosis

In the omental roll‐up group, the omentum was mobilized, brought to the posterior side of the pancreatojejunal anastomosis (Figure 2), and wrapped around to the anterior side of the pancreatojejunal anastomosis from the posterior to anterior surface of the anastomosis. The anterior side of the omentum was fixed to the pancreatojejunal anastomosis by using suture material from this anastomosis (Figure 3). In the non‐omental roll‐up group (control group), PD was performed without omental roll‐up at the pancreatic anastomosis.

### 2.4 Endpoints

The primary endpoint was the incidence of clinically relevant postoperative pancreatic fistula (POPF) by using the definition of the International Study Group on Pancreatic Fistula.^6^ The secondary endpoints were specific postoperative pancreatic complications, including postoperative hemorrhage from pancreatectomy (PPH),^7^ delayed gastric emptying (DGE),^8^ postoperative chyle leakage,^9^ and 30‐day mortality.

### 2.5 Statistical analysis

The sample‐size determination was based on the estimate of a previous retrospective study to detect a two‐sided difference.^4^ Using clinically relevant pancreatic fistula of 35.0% in non‐omental roll‐up group and 3.4% in omental roll‐up group (α = 0.05, power 80%), it was calculated that 30 patients per arm would be required. Statistical analysis was carried out using SPSS software version 20 (IBM Corp., Armonk, NY, USA). Variables were compared using the χ test and independent samples *t*‐test. Differences were considered significant at a *P*‐value of < 0.05. Univariate and multivariate logistic regression analyses were performed by the stepwise technique.

## 3 RESULTS

### 3.1 Patients’ characteristics

A total of 68 patients were included in this study. The patients’ demographics are summarized in Table 1. There were no differences in age, sex, body mass index, and clinical presentation between the two groups. For histological diagnosis, a significantly higher incidence of pancreatic cancer was found in the omental roll‐up group than in the control group (*P* = 0.042). No significant difference in the operative procedure was observed between the two groups, but the rate of the classical Whipple operation tended to be higher in the omental roll‐up group than in the control group (*P* = 0.073). No significant difference in preoperative biliary drainage was found between the two groups. Preoperative laboratory data, including total bilirubin, albumin, and alkaline phosphatase levels, as well as pancreatic fistula risk classification,^10^ were not significantly different between the two groups. No significant difference in the median tumor size was found between the two groups. Pancreatojejunal anastomosis was frequently performed using the Modified Blumgart technique in both groups, with no difference in incidence between the groups. Short internal stenting was commonly used for pancreatojejunal anastomosis and its incidence was similar between the groups. Postoperative somatostatin was used in a few patients in each group.

**TABLE 1:** Patients’ characteristics

	Non‐omental roll‐up (n = 34)	Omental roll‐up (n = 34)	*P*‐value
Age (years), mean ± SD	58.2 ± 11.14	62.0 ± 10.91	0.164
Sex, n (%)			
Male	16 (47.1)	17 (50.0)	
Female	18 (52.9)	17 (50.0)	0.808
Body mass index (kg/m^2^), mean ± SD	23.2 ± 3.85	22.7 ± 5.15	0.698
Presentation, n (%)			
Incidental	3 (8.8)	3 (3.8)	
Jaundice	20 (58.8)	22 (64.7)	
Abdominal pain	6 (17.6)	4 (11.8)	
Weight loss	2 (5.9)	1 (2.9)	
Others	3 (8.8)	4 (11.8)	0.914
Histological diagnosis, n (%)			
Pancreatic cancer	9 (26.4)	15 (44.1)	
Cholangiocarcinoma	3 (8.8)	2 (5.9)	
Ampullary cancer	10 (29.4)	6 (17.7)	
Duodenal cancer	1 (2.9)	2 (5.9)	
IPMN	1 (2.9)	0	
PNET	3 (8.8)	3 (8.8)	
Others	7 (20.6)	6 (17.7)	0.042
Operation, n (%)			
Classical Whipple	19 (55.9)	26 (76.5)	
PPPD	15 (44.1)	8 (23.5)	0.073
Preoperative biliary drainage, n (%)	21 (61.8)	24 (70.6)	0.442
Total bilirubin (mg/dL), median (range)	0.8 (0.6‐2.9)	1.45 (0.8‐2.2)	0.142
Albumin (g/dL), mean ±SD	33.8 ± 7.17	34.5 ± 4.08	0.593
ALP, median (range)	150.5 (97‐316)	142.5 (86‐291)	0.796
Pancreatic duct (mm), median (range)	3.0 (2‐5)	4.0 (3‐6)	0.195
Pancreatic texture, n (%)			
Soft	20 (58.8)	15 (44.1)	
Firm	7 (20.6)	9 (26.5)	
Hard	7 (20.6)	10 (29.4)	0.474
Pancreatic fistula risk, n (%)			
Low risk	8 (23.5)	15 (44.1)	
Intermediate risk	21 (61.8)	16 (47.1)	
High risk	5 (14.7)	3 (8.8)	0.199
Tumor size (cm), median (range)	2.6 (2.1‐4.5)	2.7 (1.9‐4.0)	0.728
Pancreatoenteric anastomosis, n (%)			
Modified Blumgart	20 (58.8)	26 (76.5)	
Cattle‐Warren	11 (32.4)	5 (14.7)	
Invagination	2 (5.9)	2 (5.9)	
Telescopic	1 (2.9)	1 (2.9)	0.343
Stenting, n (%)			
No stent	8 (23.5)	12 (35.3)	
Short internal stent	15 (44.1)	17 (50.0)	
External stent	11 (32.4)	5 (14.7)	0.204
Vascular resection, n (%)	3 (8.8)	9 (26.5)	0.056
Postoperative somatostatin, n (%)	2 (5.9)	4 (11.7)	0.673
ASA, n (%)			
Class I/II	17 (50.0)	18 (54.5)	
Class III/IV	17 (50.0)	15 (45.5)	0.808

Abbreviations: ALP, alkaline phosphatase; ASA, American Society of Anesthesiologists; IPMN, intraductal papillary mucinous neoplasm; PNET, pancreatic neuroendocrine tumor; PPPD, pylorus‐preserving pancreaticoduodenectomy.

### 3.2 Surgical outcomes

The surgical outcomes are summarized in Table 2. There was one death in the control group due to myocardial infarction. The incidence of major complications and clinically relevant POPF was not different between the two groups. There were also no significant differences in PPH, DGE, and chyle leakage between the two groups. Furthermore, the operative time and the length of hospital stay were similar in the two groups. The surgical margin rate of R0 was significantly higher in the control group than in the omental roll‐up group (*P* = 0.023). The omental roll‐up group had one PPH case. This patient developed POPF and the PPH occurred on postoperative day 16. The patient was then taken to the operating room and the bleeding site was at gastroduodenal artery (GDA) stump, which was inflamed and friable resulting from POPF.

**TABLE 2:** Outcomes of treatment

	Non‐omental roll‐up (n = 34)	Omental roll‐up (n = 34)	*P*‐value
Major complication, n (%)	14 (41.2)	12 (35.3)	0.618
Clinically relevant pancreatic fistula, n (%)	7 (20.6)	5 (14.7)	0.525
Post‐pancreatectomy hemorrhage, n (%)	1 (2.9)	1 (2.9)	1.0
Delayed gastric emptying, n (%)	4 (11.8)	3 (8.8)	0.690
Chyle leakage, n (%)	1 (2.9)	5 (14.7)	0.197
Postoperative intervention, n (%)	10 (29.4)	4 (11.8)	0.072
Type of complication, n (%)			
Incisional SSI	6 (17.6)	9 (26.5)	
Organ/space SSI	4 (11.8)	1 (2.9)	
Others	4 (11.8)	5 (14.7)	0.469
Operative time (min), mean ± SD	486.2 ± 116.7	459.9 ± 114.5	0.354
Blood loss (mL), median (range)	500 (300‐800)	600 (300‐1000)	0.515
Length of hospital stay (days), median (range)	17.5 (11.0‐31.0)	15 (11.0‐25.0)	0.359
Margin, n (%)			
R0	32 (94.1)	24 (70.6)	
R1	2 (5.9)	9 (26.5)	
R2	0	1 (2.9)	0.023
Postoperative WBC count, mean ± SD			
Day3	11 579 ± 4462	12 105 ± 3851	0.604
Day 14	10 105 ± 6045	11 729 ± 19 278	0.641
Readmission, n (%)	1 (2.9)	2 (5.9)	0.314

Abbreviations: SSI, surgical site infection; WBC, white blood cell.

Age, operative procedure, pancreatic fistula risk classification, pancreatic stenting, vascular resection, and omental roll‐up were included in univariate logistic regression analysis to identify risk factors of POPF. However, no factors were significantly related to the occurrence of POPF (Table 3).

**TABLE 3:** Univariate and multivariate logistic regression analyses of postoperative pancreatic fistula risk factors

	OR (95% CI)	*P*‐value
Age (years)	1.01 (0.96, 1.07)	0.509
Omental roll‐up		
Yes	0.67 (0.18,2.34)	0.526
No	1	–
Operation		
PPPD	3.50 (0.96, 12.6)	0.056
Classical Whipple	1	–
Pancreatic fistula risk		
High	3.50 (0.40, 30.3)	0.256
Intermediate	2.89 (0.55, 15.05)	0.206
Low	1	–
Stenting		
Internal	3.51 (0.37, 32.58)	0.268
External	1.39 (0.20, 10.29)	0.134
No	1	–
Vascular resection		
Yes	0.37 (0.04, 3.19)	0.367
No	1	–
Pancreatic duct (mm)	0.89 (0.67, 1.18)	0.442
Total bilirubin (mg/dL)	0.94 (0.77, 1.14)	0.550

Abbreviations: CI, confidence interval; OR, odds ratio; PPPD, pylorus‐preserving pancreaticoduodenectomy.

## 4 DISCUSSION

**FIGURE 4:**
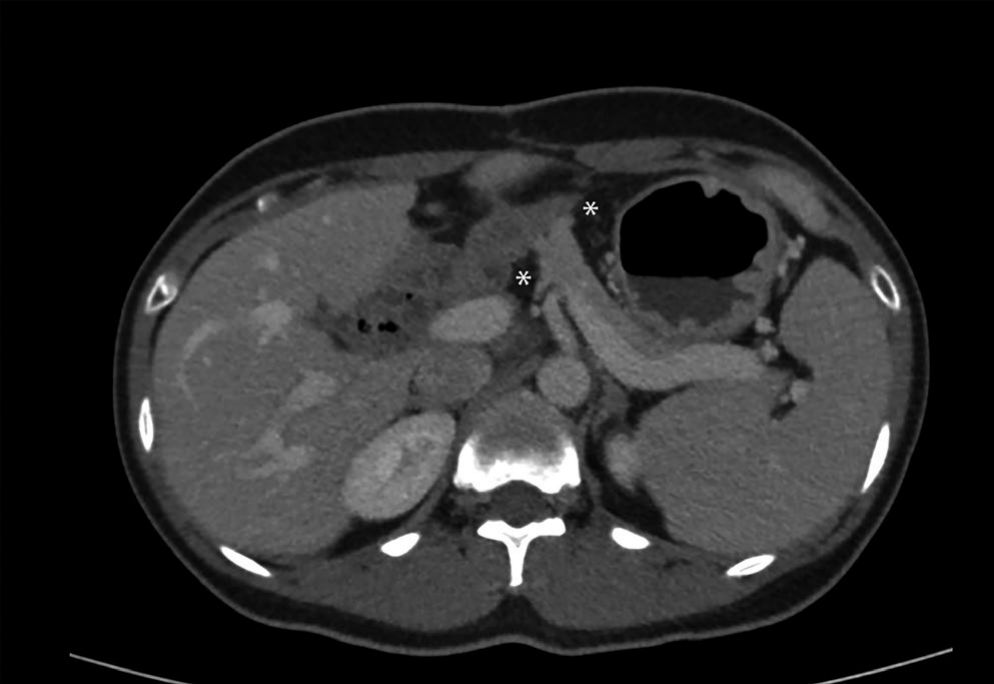
Computed tomography scan shows omentum around pancreatojejunal anastomosis without portal vein compression. *Omentum

The PD procedure is associated with the incidence of POPF, which remains an important complication, with an incidence of approximately 16.7%‐23.4%.^1^, ^11^ Omental coverage uses an autologous tissue, is an easily applicable procedure, and there is no further cost during PD. Retrospective studies have investigated tissue coverage in pancreatoenteric anastomosis.^4^, ^5^, ^12^, ^13^, ^14^, ^15^ However, for reducing POPF, the omental roll‐up procedure is still controversial. Many previous studies have reported that omental roll‐up or a round ligament flap reduces the rate of POPF after pancreatojejunal reconstruction or pancreatogastric reconstruction.^4^, ^13^, ^15^ However, a reduction in POPF was not found in other studies.^5^, ^12^ A systematic review and a meta‐analysis of 14 retrospective studies of omental or falciform ligament wrapping in PD showed no benefits of the tissue wrapping procedure in minimizing the risk of POPF.^16^, ^17^ This is the first RCT to prove the benefit of the omental roll‐up procedure in reducing POPF. However, there was no benefit after using omental roll‐up and no direct complications related to the omental roll‐up procedure. Possible complications from the omental roll‐up procedure are intestinal obstruction, omental flap necrosis, omental compression of the portal vein or superior mesenteric vein (SMV), DGE, and abdominal collection.^18^ In the present study, there was no omental infarction or portal vein compression as shown by a follow‐up CT scan 6 months postoperatively (Figure 4). To prevent these complications, the omental flap should be properly tailored, not too thick, tension‐free, and have a well‐preserved vascular supply. The round ligament flap wrapping at pancreatojejunal anastomosis was reported in one study.^13^ The limitations of round ligament flap when compared to omental flap are: (a) the blood supply of the round ligament flap is not well‐demonstrated, unreliable,^19^ and might be the cause of a flap necrosis; and (b) the round ligament flap isn't long enough to wrap around pancreatojejunal anastomosis in all patients.^13^ For these reasons, omental flap is chosen to wrap around pancreatojejunal anastomosis instead of round ligament flap.

The omental roll‐up group had one PPH case, and the bleeding site was at GDA stump, which was inflamed and friable resulting from POPF. In our opinion, the pancreatic juice could still escape from omental wrapping and erode GDA stump. This is consistent with the data of the systematic review and meta‐analysis that the omental wrapping in PJ anastomosis didn't reduce the rate of PPH.^20^ Another technique of tissue flap is wrapping to skeletonized vessels including common hepatic artery, proper hepatic artery, and stump of GDA to prevent PPH. This technique has been only reported in retrospective studies and it was shown that the incidence of PPH is lower after omental flap coverage.^12^, ^14^ RCT studies need to be conduct in future.

The incidence of surgical site infection (SSI) at our institute has been slightly high due to the high rate of preoperative biliary drainage, which is a risk factor of SSI. Moreover, cefoxitin was used as a preoperative prophylactic antibiotic and was continued for 24 hours postoperatively in our protocol. This might not cover pathogen of SSI.^21^ The trial of broad‐spectrum antibiotic to reduce the incidence of SSI was now conducted and assessed under data collecting in our hospital. The incidence of pancreatic cancer was significantly higher and there was a more positive margin in the omental roll‐up group than in the control group. The difference in histological diagnosis between the two groups might have occurred incidentally and be from the low sample size in this trial. Because there was only one previous study that had the same technique of omental roll‐up as ours, the sample size was calculated by only one study that might be underestimated and underpower RCT.

## 5 CONCLUSION

This study shows that omental roll‐up does not decrease the incidence of POPF after PD.

## CONFLICT OF INTEREST

Nothing to declare.
